# Functional Electrical Stimulation of Intrinsic Laryngeal Muscles under Varying Loads in Exercising Horses

**DOI:** 10.1371/journal.pone.0024258

**Published:** 2011-08-31

**Authors:** Jon Cheetham, Abby Regner, Jonathan C. Jarvis, David Priest, Ira Sanders, Leo V. Soderholm, Lisa M. Mitchell, Norm G. Ducharme

**Affiliations:** 1 Department of Clinical Sciences, College of Veterinary Medicine, Cornell University, Ithaca, New York, United States of America; 2 Institute for Ageing and Chronic Disease, University of Liverpool, Liverpool, United Kingdom; 3 Hackensack University Medical Center, Hackensack, New Jersey, United States of America; Harvard Medical School, United States of America

## Abstract

Bilateral vocal fold paralysis (BVCP) is a life threatening condition and appears to be a good candidate for therapy using functional electrical stimulation (FES). Developing a working FES system has been technically difficult due to the inaccessible location and small size of the sole arytenoid abductor, the posterior cricoarytenoid (PCA) muscle. A naturally-occurring disease in horses shares many functional and etiological features with BVCP. In this study, the feasibility of FES for equine vocal fold paralysis was explored by testing arytenoid abduction evoked by electrical stimulation of the PCA muscle. Rheobase and chronaxie were determined for innervated PCA muscle. We then tested the hypothesis that direct muscle stimulation can maintain airway patency during strenuous exercise in horses with induced transient conduction block of the laryngeal motor nerve. Six adult horses were instrumented with a single bipolar intra-muscular electrode in the left PCA muscle. Rheobase and chronaxie were within the normal range for innervated muscle at 0.55±0.38 v and 0.38±0.19 ms respectively. Intramuscular stimulation of the PCA muscle significantly improved arytenoid abduction at all levels of exercise intensity and there was no significant difference between the level of abduction achieved with stimulation and control values under moderate loads. The equine larynx may provide a useful model for the study of bilateral fold paralysis.

## Introduction

Bilateral vocal fold paralysis (BVCP) is a life threatening condition. Normally the vocal folds open during inspiration and close during swallowing, however, during BVCP the vocal folds become immobile in a closed position and can cause airway obstruction [1]–[3]. In most cases of BVCP, some innervation is present and although it is insufficient to naturally open the vocal folds, electrical stimulation can usually evoke the small amount of vocal fold opening needed to prevent airway obstruction [4]. As a result, this condition appears to be a good candidate for therapy using functional electrical stimulation (FES). Due to the relatively inaccessible location and small size of the sole arytenoid abductor, the posterior cricoarytenoid muscle, developing a working FES system has been technically difficult.

A naturally-occurring disease in horses shares many functional and etiological features with BVCP in humans. In horses, recurrent laryngeal neuropathy (RLN), results in demyelination of the recurrent laryngeal nerve with axonal death [5]–[7] and produces a mixed denervation/reinnervation synkinetic pattern similar to that seen in patients with BVCP following injury to the recurrent laryngeal nerve [8]–[11]. Functionally, this leads to atrophy of the posterior cricoarytenoid muscle reducing its ability to dilate the rima glottidis and leading to a relative upper airway obstruction with stridor and reduced athletic ability.

Previous animal models investigating FES in laryngeal disease have predominantly focused on the dog [4], [12], [13], sheep [14], [15],cat [16] and most recently in horses [17]. The horse has a number of advantages as a model for developing an FES system to reanimate the vocal fold as the longitudinal assessment of function is possible and the larger size of the equine PCA muscle reduces some of the technical challenges of implantation in the PCA in smaller species. This enables optimization of stimulation parameters and electrode placement prior to size reduction for clinical human implantation.

In this study the feasibility of FES for equine vocal fold paralysis was explored by testing arytenoid abduction evoked by electrical stimulation. The first aim of this study was to determine rheobase and chronaxie in innervated equine PCA muscle. The second aim was to test the hypothesis that direct muscle stimulation can maintain airway patency during strenuous exercise in horses with induced transient conduction block of the laryngeal motor nerve.

## Materials and Methods

### Experimental design

Six adult horses (Age range 6–19years, weight range 436–530 kg) with normal arytenoid function (Havemayer grade 1 [18],) were instrumented with a single bipolar intra-muscular electrode (5 French) in the left posterior cricoarytenoid (PCA) muscle. Electrical testing and stimulation were performed two weeks post operatively. All procedures were approved by Cornell University Institutional Animal Care and Use Committee (protocol 2008-0146, approved 11-17-2008).

### Surgical procedure

Horses were placed under general anesthesia in right lateral recumbency and the skin overlying the lateral aspect of the larynx and proximal trachea prepared aseptically. An inferior laryngeal approach was used. A 5 cm incision was made, centered on the inferior aspect of the cricoid cartilage immediately ventral and parallel to the linguofacial vein and the ventral border of the left sternocephalicus muscle. The omohyoideus muscle was bluntly separated from the linguofacial vein to expose the caudal aspect of the cricoid cartilage.

#### Identification of location for electrode placement

The lateral compartment of the PCA has been shown to have the most effect on vocal fold opening [10] and this region was explored for low threshold electrically evoked contraction. A split-o-can Pajunk needle (Pajunk GmbH, Geisingen, Germany) with an inner insulated guide wire was used to produce stimulation at the tip of the needle. The intramuscular location within the lateral neuromuscular compartment associated with maximal arytenoid abduction was determined by stimulation with a portable device (Stilwell, Med-El, Innsbruck, Austria) using one second bursts of 16 Hz, with a 750 µs pulse duration and amplitude of 1 to 5mA while simultaneously observing arytenoid movement with a videoendoscope placed through the left nostril into the nasopharynx.

#### Electrode placement

**Figure 1:**
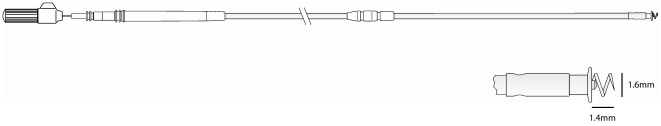
Illustration of KY5 electrode. Screw length 1.4 mm with 1.5 turns; screw diameter 1.6 mm; screw surface area 7.2 mm^2^. Screw is Eligiloy with Iridium oxide coat. Total electrode length 580 mm.

A custom made 5Fr bipolar electrode (KY5, Osypka GmbH, Medizintechnik, Rheinfelden, Germany, Figure 1) was then placed at the optimal location within the lateral neuromuscular compartment of the PCA muscle. To prevent the electrode from being dislodged from the PCA the electrode was secured to the cricoid cartilage approximately 20 mm from the electrode tip using OO nylon suture. The electrode lead was tunneled subcutaneously to a programmable internal stimulator placed in the intermandibular area. All horses received broad-spectrum antibiotics (trimethoprim-sulfadiazine 30 mg/kg, PO, BID) and Phenylbutazone (1 mg/kg, PO, BID) for 5 to 7 days. All horses were examined daily for any signs of complications or illness (pain, swelling, dysphagia etc.).

### Design of neuroprosthesis

The neuroprosthesis is a modified cochlear implant stimulator (Pulsar, Med—El Corporation, Innsbruck, Austria) with an output consisting of 12 array electrodes and a reference ground. Six of the 12 array electrodes from the internal stimulator are connected to the anode and six to the ground of the unit to the cathode with platinum iridium leads. The unit is capable of delivering biphasic pulses up to 49 Hz with a pulse duration of up to 4.27 ms and is a current-controlled device adjusted using an external computer which communicates with the internal coil through inductive coupling and magnetic fixation.

All stimulation pulses used were biphasic with the negative (cathodic) phase occurring before the positive phase. A 5 µs interphase gap was used throughout.

### Electrical Excitability and Stimulation Testing

Strength-duration curves were first obtained at rest to determine the recruitment characteristics of each animal's electrode. Threshold was defined as the smallest discernable twitch of the arytenoid cartilage. Stimulation trials were then performed in horses at exercise using pulse trains that produced maximal vocal fold abduction.

### Strength-duration trials

To determine the excitation characteristics of the equine PCA muscle, rheobase - the minimum amplitude needed to elicit a threshold response at infinitely long pulse duration of the electrical stimulation and chronaxie -the pulse duration at twice the rheobase were determined [19]. With the horse under standing sedation using detomidine hydrochloride (0.01 mg/kg IV), a videoendoscope was placed in the right ventral nasal meatus so that its tip was located at the level of the rostral part of the epiglottic cartilage. The examination was recorded on a DVD recorder for subsequent analysis. Single biphasic balanced pulses were applied to the left PCA muscle with pulse durations from 0.05 ms to 6.8 ms. Pulse amplitude was increased from zero in 0.1mA steps until a single abduction twitch of the left arytenoid cartilage was observed via the endoscope (threshold). Each stimulus was applied during expiration hold (the short hesitation at the end of expiration). Threshold was readily observed endoscopically.

### Exercise Protocol and Instrumentation

Horses were exercised using a standard protocol [20]. Briefly, horses were trained 5 days/week on a high-speed treadmill for 5 weeks before each trial to adapt them to the exercise protocol and instrumentation system and standardize their fitness level. Horses were shod with flat, aluminum shoes with toe clips. Close-fitting neoprene boots (Professional's Choice, Professional's Choice Sports Medicine Products Inc., Spring Valley, CA) were placed on the lower limbs to prevent injuries from interference with other limbs. A nylon halter capable of bearing the subjects' weight provided security from injury during treadmill examination. Horses were fasted for 3 hours before any exercise trial [21]. At the end of each exercise period, horses were bathed and cooled out appropriately.

**Figure 2:**
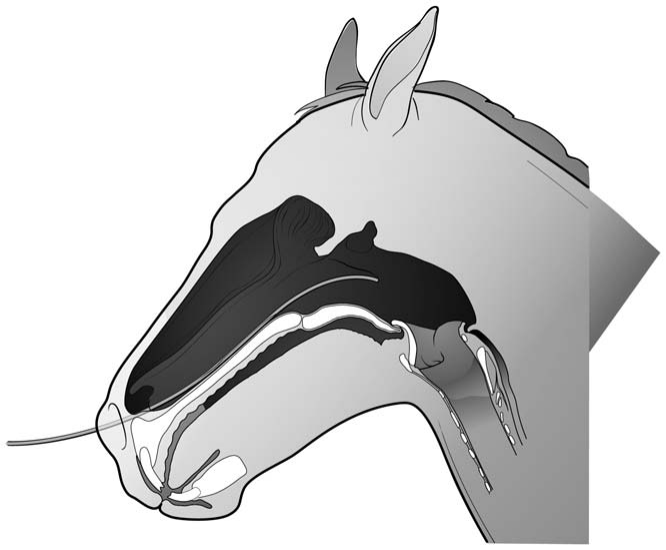
Illustration to demonstrate position of vidoendoscope in nasopharynx during resting and exercising endoscopy. Endoscope secured to horse's halter (not shown).

Data recorded during exercise trials included heart rate, electrocardiogram and accelerometer measurements. Nasopharyngeal and laryngeal movements were recorded using a flexible videoendoscope (Olympus GIF-140) passed into the nasopharynx via the right ventral nasal meatus and secured to the horse's halter (figure 2). Heart rate was measured by an on-board monitor (Hippocard Systems, Lexington, KY). Data were recorded onto DVD disks for subsequent analysis.

Each horse performed a Standardizing Fitness Test. At time 0, the treadmill was started and accelerated to 4 m/s. After 1 minute at 4 m/s, the treadmill was accelerated to 6 m/s and maintained at that speed for 1 minute, then the speed was increased to 10 m/s and held at that speed for 1 min. Each subsequent minute, the treadmill was accelerated by 1 m/s until the horse was no longer capable of maintaining its position near the front of the treadmill. The heart rate at this speed was determined to be the maximum heart rate (HRmax) [22]All horses were run under control conditions to determine that they had a normal upper respiratory tract at exercise (laryngeal grade IA) [18]. A minimum of three days was allowed for recovery between each trial. Two experimental trials were then performed with transient block of the left recurrent laryngeal nerve.

### Recurrent Laryngeal Nerve Block

Temporary vocal fold paralysis was experimentally invoked by injection of local anesthetic around the left recurrent laryngeal nerve (RLN) to produce conduction block. The horse was not sedated for this procedure. To locate the RLN a 50-mm stimulating needle (Stimuplex Insulated Needle; Braun Medical, Bethlehem, PA) was inserted just dorsal to the jugular vein in the mid cervical region and advanced perpendicular to the skin to contact the carotid sheath. The needle was advanced and stimulated at an initial output of 2 mA, a frequency of 2 Hz, and a pulse duration of 0.15 ms (Innervator 232; Fisher & Paykel Healthcare, Auckland, New Zealand) until arytenoid abduction twitches were seen endoscopically. The current was then reduced in 0.2-mA decrements and the needle advanced further until the same response was elicited. Criteria for correct final needle position included absence of blood aspiration, cessation of twitch response after injection of a test volume of mepivicaine (0.5 ml, Raj test), and no resistance to injection [23]. If these criteria were met, the remainder of the mepivicaine (4.5 ml) was administered slowly. The horse was allowed to rest for 30 min and then evaluated on the treadmill.

### Frequency response trial

**Figure 3:**
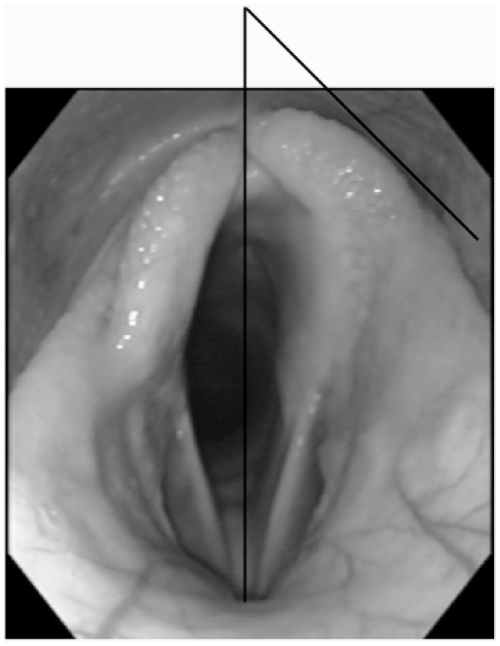
Method measuring arytenoid abduction. Briefly a line is drawn connecting the dorsal- and ventral-most points of the *rima glottidis.* This line is then extended dorsally for a distance of one third of the dorsoventral height of the *rima glottidis*. A tangential line to the arytenoid cartilages is drawn, and the angle between the dorsoventral line and the tangential line is measured.

The relationship between stimulation frequency and pulse duration was investigated at 90% HR_max_. The stimulation unit was used to deliver each of six combinations of frequency and pulse duration. Three frequencies (15, 24 and 49 Hz) and two pulse durations (0.427 ms and 4.27 ms) were used. A pulse amplitude of 6mA was used throughout. These pulse durations were selected to provide an order of magnitude increase. The sequence of each combination of pulse duration and frequency was randomized and applied for 20 seconds with a 5 second gap between stimulation periods. For each combination of pulse duration and frequency still images of the *rima glottidis* were captured during inspiration from the DVD recordings using software (Video Wizard, Womble Multimedia, 20333 Bollinger Road Cupertino, CA, USA). The degree of arytenoid abduction was measured using a previously validated technique [24] with commercially available software (Able Image Analyser) ^3^. Briefly a line is drawn connecting the dorsal- and ventral-most points of the *rima glottidis.* This line was then extended dorsally for a distance of one third of the dorsoventral height of the *rima glottidis*. A tangential line to the arytenoid cartilages was drawn, and the angle between the dorsoventral line and the tangential line measured (figure 3).

### Increasing exercise intensity trial

The relationship between exercise intensity and arytenoid function was also determined. Horses were exercised for 60 seconds at 50, 80, 90 and 100% HR_max_ under control conditions (no block, no stimulation) and the degree of arytenoid abduction determined as described above. On a separate occasion, transient recurrent laryngeal nerve dysfunction was induced and the horses exercised for 60 seconds at 50, 80, 90 and 100% HR_max_. For the first 30 seconds of each interval no stimulation was applied (block) and in the second 30 seconds of each interval stimulation was applied (block+stimulation) (4.27 ms pulse duration, 49 Hz). The maximal output of the unit (6mA) was used for stimulation. Evidence of aspiration was assessed endoscopically following each exercise trial.

### Data Analysis

Threshold (mean ± standard error) was determined for each pulse duration. For the frequency-response trials, a mixed effect model was fitted to the data to determine the relationship between left arytenoid abduction and horse as a random effect, pulse duration (0.427 ms or 4.27 ms), frequency (15,24,49 Hz) and trial position in the randomized stimulation sequence (1–6) as fixed effects and an interaction term for pulse duration*frequency. A similar model was fitted to determine the relationship between the degree of arytenoid abduction, exercise intensity (50, 80, 90 and 100% HR_max_) and condition of the posterior cricoarytenoid muscle (control, induced motor block, induced motor block with stimulation). Tukey's *post hoc* tests and linear contrasts were used as appropriate. Statistical analysis was performed using JMP (SAS Institute, Cary, North Carolina, USA). Significance was set at p<0.05 throughout.

## Results

All horses recovered uneventfully from surgery. Postoperative swelling and discomfort were minimal and resolved within 10–14 days. Appropriate healing was obtained in all horses at suture removal. Horses remained comfortable throughout the study.

**Figure 4:**
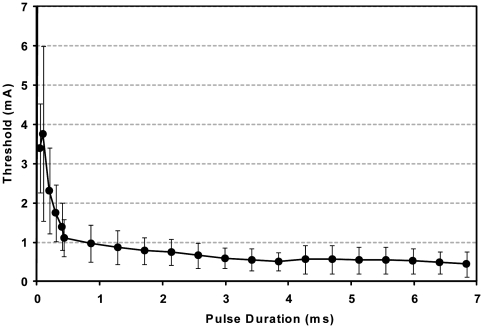
Strength-duration curve for innervated equine posterior cricoarytenoid muscle at rest. Data shown are mean and standard deviation for six horses.

The relationship between pulse duration and the threshold stimulus required to elicit a visible twitch of the arytenoid followed a typical pattern for innervated muscle (Figure 4). Rheobase was determined as 0.55±0.38 v with chronaxie 0.38±0.19 ms.

**Figure 5:**
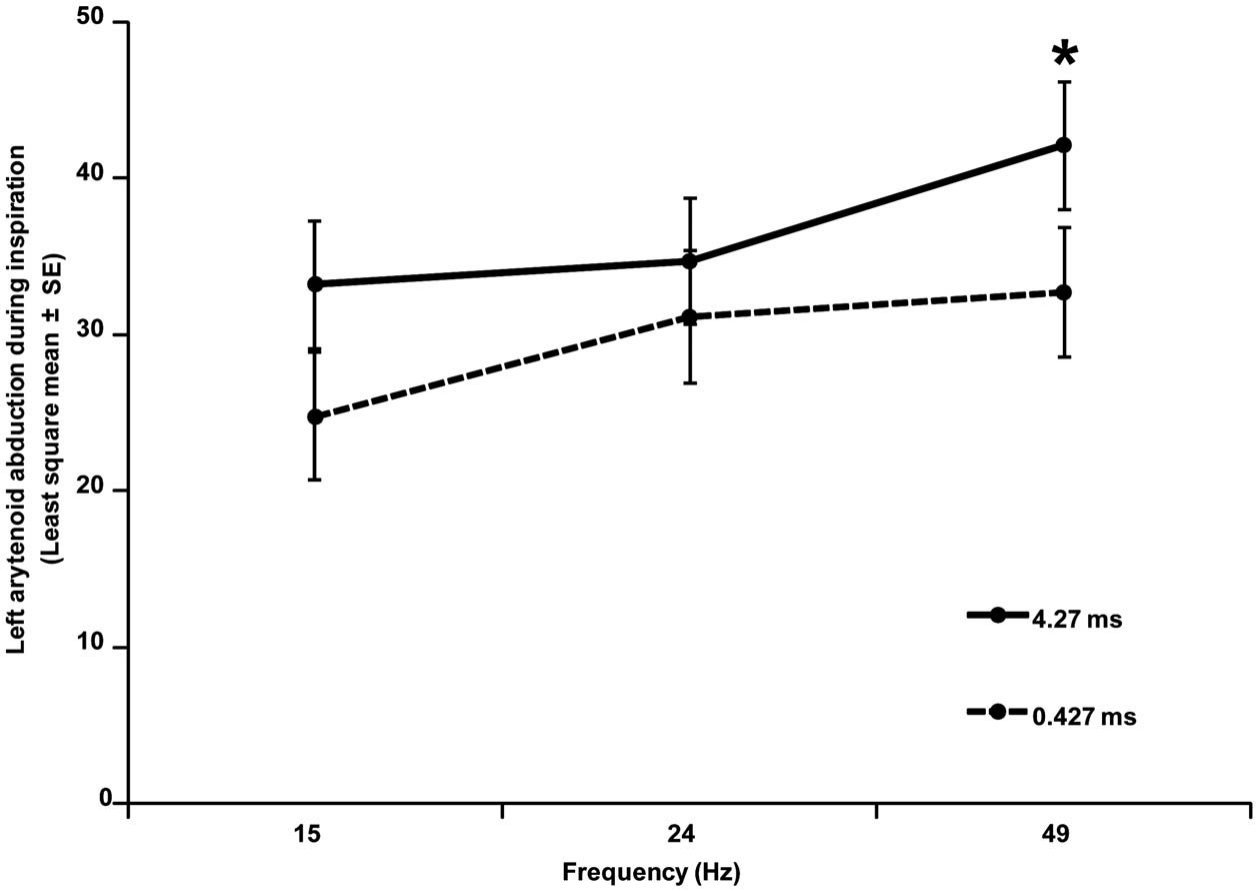
Titration of pulse duration and stimulation frequency with left abduction of the left arytenoid for horses with induced motor conduction block at 90% HRmax demonstrating significantly more abduction with longer pulse duration (p = 0.0037) and higher frequency (p<0.002). Linear contrast (*) indicates increased abduction at 49 Hz with pulse duration of 4.27 ms versus 0.427 ms (p = 0.03).

Recurrent laryngeal nerve block produced transient dysfunction of the left recurrent laryngeal nerve with complete paralysis of the muscles acting on the left arytenoid cartilage in all horses. Stimulation frequency and pulse duration both had significant effects on the angle of abduction of the left arytenoid (Figure 5). Functional electrical stimulation of the PCA muscle did not produce signs of discomfort or pain in any horse. Controlling for other factors in the model, a longer pulse duration (4.27 ms, mean ± SEM, 36.6° ±3.52°) produced significantly more abduction than a shorter pulse duration (0.427 ms, 29.6° ±3.6°, p = 0.0037). A frequency of 49 Hz produced significantly more left arytenoid abduction than the other two levels (15 Hz, 29.2° ±3.6°; 24 Hz, 31.3° ±3.7°; 49 Hz, 38.9° ±3.7°, p<0.002). There was no significant effect of trial position in the sequence of stimulation (p = 0.069). The interaction term (frequency*pulse duration) was not significant, however, a linear contrast demonstrated significantly more abduction at 49 Hz with a pulse duration of 4.27 ms than at 49 Hz with a pulse duration of 0.427m s. Overall model fit was good (Adjusted R^2^ = 0.85).

**Figure 6:**
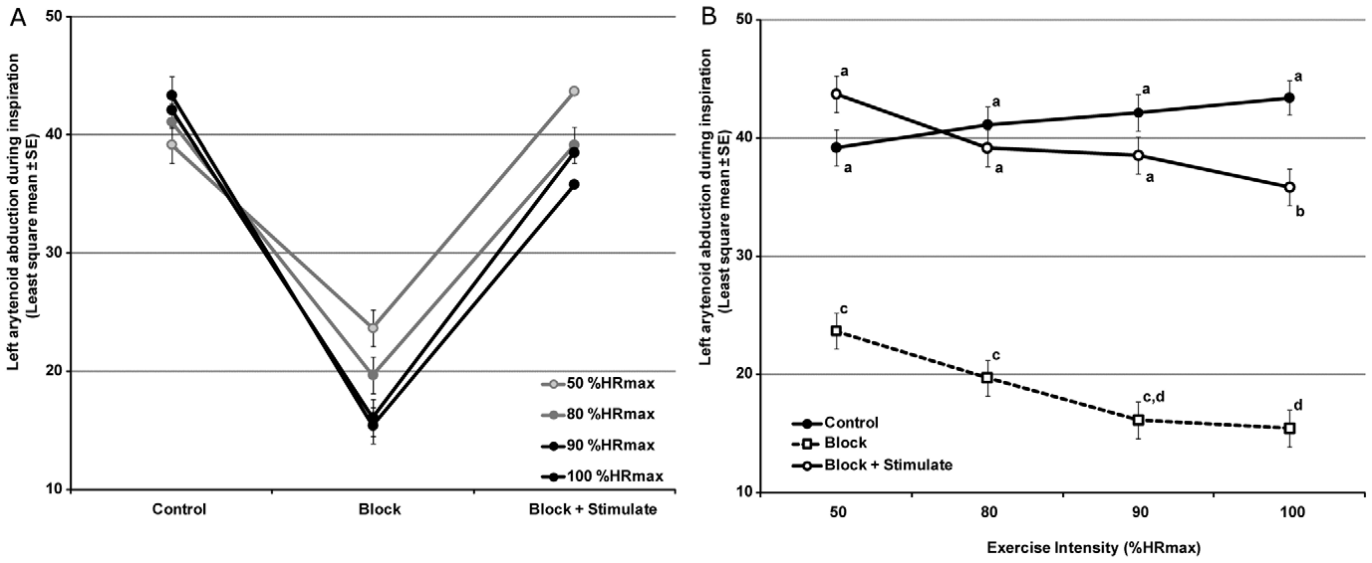
Relationship between stimulation and arytenoid abduction during inspiration at increasing loads (exercise intensity). (A) Increasing load produced increased abduction under control conditions but decreased abduction with block and block+stimulation. Stimulation at 4.27 ms pulse duration, 49 Hz, 6mA. Data shown are Least Squares (Adjusted) means with standard error. (B) Same data plotted to demonstrate restoration of function by stimulation at moderate exercise intensities (50–90%HRmax). Letters denotes significant differences with Tukey's *post hoc* test at p<0.05.

Increasing exercise intensity produced increased left arytenoid abduction in control trials and decreasing arytenoid abduction in blocked/un-stimulated trials (Figure 6a). No evidence of aspiration was observed in any trial. Controlling for other factors in the model, left arytenoid abduction under control conditions at 50,80,90 and 100% HR_max_ was 39.2° ±1.5°, 41.1° ±1.5°, 42.1° ±1.5° and 43.4° ±1.4° respectively (Figure 6b). With transient recurrent laryngeal nerve block, left arytenoid abduction at the same exercise intensity levels was significantly lower at 23.6° ±1.5°, 19.7° ±1.5°, 16.1° ±1.5° and 15.4° ±1.5° respectively (p<0.001). Intramuscular stimulation of the posterior cricoarytenoid muscle significantly improved arytenoid abduction at all levels of exercise intensity –left arytenoid abduction was 43.7° ±1.5°, 39.2° ±1.5°, 38.6° ±1.5° and 35.9° ±1.5° respectively (p<0.001). The interaction term (%HR_max_*muscle status (control, block, block+stimulate)) was significant (p<0.0007). Controlling for other factors in the model there was no significant difference between the level of abduction achieved with stimulation and control values at 50, 80 and 90% HR_max_. Abduction with stimulation at 100%HRmax was significantly lower than control values (p<0.05). Overall model fit was good (Adjusted R^2^ = 0.90).

## Discussion

This study demonstrates that intra-muscular stimulation of the posterior cricoarytenoid muscle can maintain airway patency during strenuous exercise in animals with induced transient laryngeal paralysis. Testing under those conditions is a stringent outcome measure for reanimation of the larynx. Indeed, exercise in horses subjects the arytenoid to very negative inspiratory pressures and is likely a more strenuous requirement than restoration of abduction in humans, or other preclinical models at rest [13], [25], [26]. Although there was no significant difference in arytenoid abduction at 50, 80 and 90% of HRmax, the response was significantly lower at 100% HRmax. This may reflect both the increasing inspiratory negative load on the left arytenoid cartilage at maximal exercise. It may also be partially due to collapse of the left vocal cord at high airflows which can produce medial deviation of the arytenoid.

Although transient recurrent laryngeal function was induced for the exercise trials, laryngeal function was normal prior to recurrent laryngeal nerve block and so the function of the motor nerve distal to the block, and function of the motor end plates was anticipated to be normal. Once the endplates are no longer functional the sarcolemma has to be stimulated directly, requiring higher stimulation parameters. Further work will be required to determine the muscle response of the equine PCA muscle in laryngeal dysfunction that usually involves nerve degeneration and muscle atrophy.

In other animal species, endoscopic observation of laryngeal function requires sedation although this has been shown to significantly reduce arytenoid abduction [27]. In the horse, arytenoid function is of high clinical importance and assessment of arytenoid abduction has become a standard technique. This procedure can be performed without sedation in the conscious animal [28]–[32]. This adaptation depends on the normal function of a highly adapted upper airway: the long axis of the larynx is rotated though 90° to optimize airflow [33] which can reach up to 80 L/s when racing at speeds of up to 44miles/hour [34]–[38]. By comparison, peak inspiratory flow in exercising humans is 2 L/s [39]. Such extreme adaptation is useful as an experimental model as it places high stresses on equine laryngeal function and so small abnormalities can be readily detected using endoscopy [40]. As a result, the likelihood of identifying functional recovery following laryngeal injury and subsequent treatment is high. This is in contrast to other species in which recovery may only be judged histologically, electromyographically or using endoscopy under general anesthesia which is known to suppress function.

Much of the literature on the effects of long-term denervation of mammalian skeletal muscle has focused on experimental studies of total sciatic section in the rat [41]–[43]. However, rat hind limb muscles enter the degenerative phase of the denervation response within a few months of injury [44] and so rat may not be a suitable model for the slower atrophy phase seen in humans. This has lead to the use of other animal models to study FES in denervated skeletal muscle [45]–[48]. The equine larynx may offer some advantages for the study of laryngeal paralysis in humans. Laryngeal paralysis occurs slowly naturally and unilaterally, providing a contralateral contol in each horse. The pathophysiology of the disease is well described due to its high importance to the equine industry [6], [10], [40], [49]–[58]. Finally, functional outcome assessment is possible using upper airway endoscopy in resting or exercising horses.

In this study a functional electrical stimulation system to reanimate the vocal fold was successfully tested in an equine model. We propose that the equine larynx is a useful model for developing laryngeal implants and electrode implantation patterns for BVCP as its larger size facilitates implantation and the assessment of function over time is possible using videoendoscopy at rest and exercise [59]. Following the characterization of appropriate stimulation parameters for denervated or synkinetically innervated muscle, implants could be reduced in size for human implantation, for example by using to 3 French electrodes.
